# Profiling of glycosylation gene expression in CHO fed-batch cultures in response to glycosylation-enhancing medium components

**DOI:** 10.1186/1753-6561-7-S6-P99

**Published:** 2013-12-04

**Authors:** Ryan Boniface, Jeoffrey Schageman, Brian Sanderson, Michael Gillmeister, Angel Varela-Rohena, John Yan, Yolanda Tennico, Shawn Barrett, Robert Setterquist, Stephen Gorfien

**Affiliations:** 1 Life Technologies Corporation, 3175 Staley Road, Grand Island, New York, USA, 14072; 2 Life Technologies Corporation, 2130 Woodward, Austin, Texas, USA, 78744; 3 Life Technologies Corporation, 29851 Willow Creek, Eugene, Oregon, USA, 97402

## Introduction

Characterization of the glycosylation profile of a recombinant protein product is an important part of defining product quality in the bioproduction industry. Development of a protein with desired characteristics would require the capacity to modify and target specific glycosylation patterns as well as an understanding of the implications of changes to these glycosylation profiles. Previous cell culture studies have demonstrated the ability to modulate glycan profiles without negative impact to culture growth and product titer through the addition of glycosylation-enhancing medium components. With new methods, including increased measurement sensitivity and new capabilities in RNA-Seq technology, it is possible to develop a glycosylation gene expression profile for CHO cells. Specific glycosylation genes can then be tracked to ensure that the addition of these compounds will not negatively impact gene expression. Analyses comparing growth and titer, glycan distribution, and transcriptome differences can present us with potential insight into what changes are taking place on a genetic level in the cell in response to changes in medium and culture conditions.

## Materials and methods

(All Materials were from Life Technologies unless otherwise indicated)

### Cell culture

CHO-S^® ^and DG44 derived recombinant cells expressing the same IgG molecule were grown in CD FortiCHO™ medium supplemented with 4mM L-glutamine and 1:100 Anti-Clumping Agent.

### Fed-batch bioreactor

DASGIP bioreactor with 500mL initial working volume seeded at 0.3x10^5 ^viable cells/ml in CD FortiCHO™ medium. 10% CD EfficientFeed™ C (EFC) feeding on days 3, 5 and 7 for CHO-S^® ^cultures, and feeding on days 4, 6 and 8 for DG44 cultures. Glucose concentration was maintained above 3g/L. Component A and/or component B were added on the first day of feeding (day 3 for CHO-S^® ^and day 4 for DG44 cultures). Culture conditions were maintained as follows; pH 7.0 +/- 0.05, 50% DO, 37°C, 110 rpm. Cell densities and viabilities were measured using a Vi-CELL^® ^counter (Beckman Coulter). Metabolites (glucose, ammonia, lactate) and IgG were measured using a Cedex^® ^Bio HT Instrument (Roche).

### Glycan analysis

Protein supernatant samples were collected and purified using POROS^® ^MabCapture^® ^A resin. Samples glycan profiles were analyzed on an Applied Biosystems^® ^3500 Series Genetic Analyzer.

### Transcriptome analysis

RNA was extracted at several time points during the culture. A total of 174 potential glycosylation specific gene targets were identified and primers designed to these using reference sequences from Chinese hamster ovary, mouse, rat and human. A total of 34 samples were multiplexed on a Proton™ PI chip on the Ion Torrent™ PGM™.

## Results and discussion

**Figure 1:**
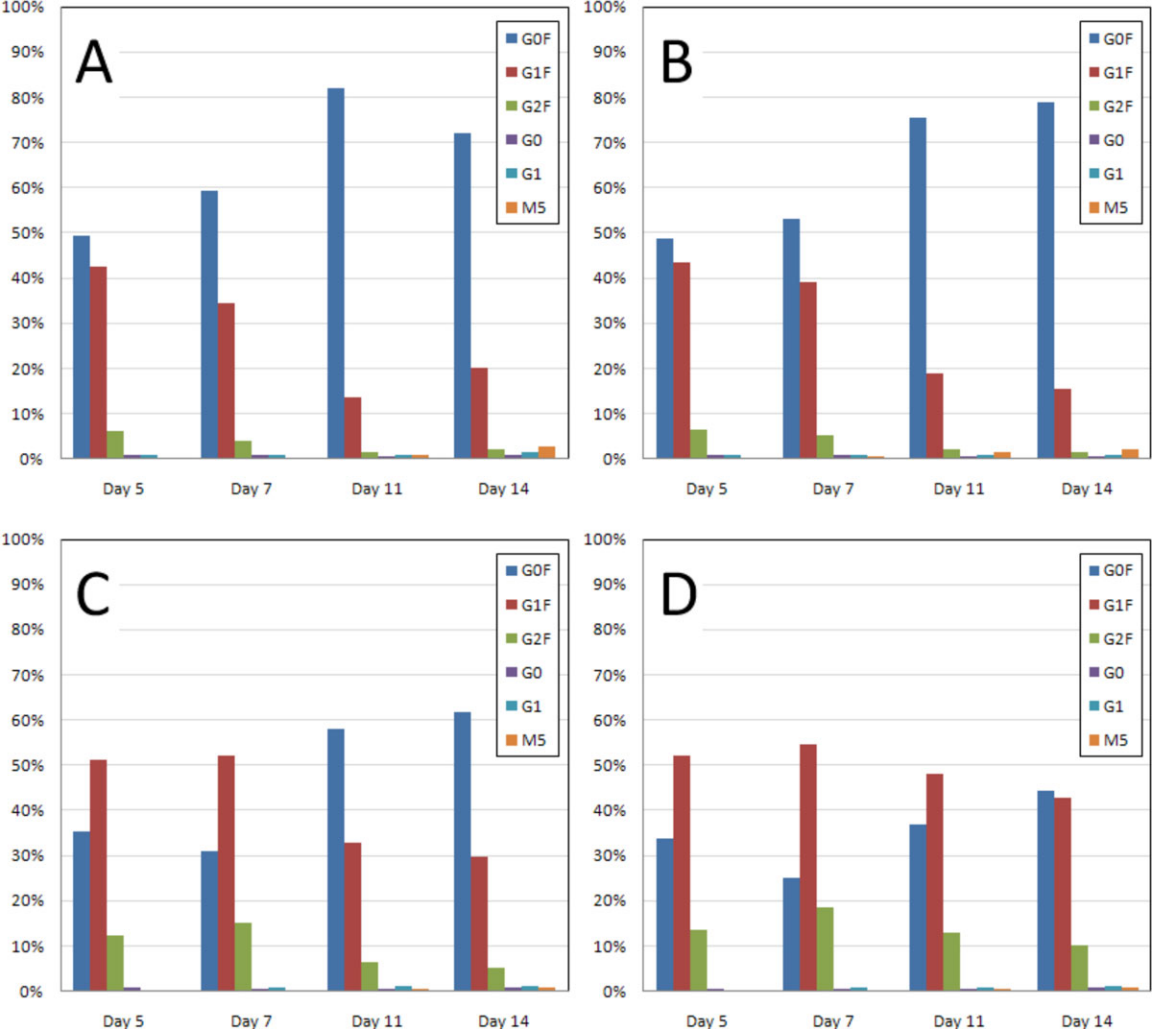
**Glycan analysis data measured as the percentage of total glycans**. (A) The glycan profile for the CHO-S^® ^culture with no addition of components A and/or B. (B) These data indicate that the addition of component A to the culture results in very little change to the glycan profile, only slight increase in the percent of G1F on days 5 and 7. (C) The addition of component B to the culture shifts the glycan profile from primarily non-galactosylated G0F to increased G1F (single galactose) and G2F (two galactose) glycoforms. (D) The addition of both components A and B results in a change in galactosylation indicated by the increase in both G1F and G2F and an overall reduction of G0F. In every condition, G0F increases with time but this is minimized with the addition of both components A and B. The majority of protein glycoforms within this experiment are fucosylated and the addition of components A and/or B does not appear to alter this.

The use of components A and B with CHO-S^® ^cells in CD FortiCHO™ medium causes a considerable increase in the level of galactosylation of the recombinant IgG (Figure 1) as shown by the shift in the glycosylation profile from G0F to G1F and G2F. The use of targeted transcriptome analysis revealed that the changes observed in the glycosylation profile do not translate to noticeable differences in the expression levels of the glycosylation genes. There are changes in gene expression levels with culture age but they are not altered by the additions of components A and/or B. It was originally theorized that components A and B could act as cofactors or substrates within the glycosylation enzymatic pathways but this could not be confirmed without an understanding of the glycosylation gene profile. The changes in the glycosylation patterns combined with the absence of changes in the gene expression data lend support to this theory. With this information it is apparent that the additions of the glycosylation-enhancing components A and B can increase galactosylation of recombinant proteins with no negative effect on growth, titer or glycosylation gene expression.

The comparison between CHO-S^® ^and DG44 cultures without supplementation with components A or B revealed the DG44 culture had better galactosylation with increased proportions of G1F and G2F. Both cell lines express high levels of DDOST, RPN1, DAD1 and SST3A which are all part of the oligosaccharyltransferase complex which catalyzes the transfer of high mannose oligosaccharides from lipid-linked oligosaccharide donors to the asparagines on the Asn-X-Ser/Thr of the polypeptide chain. The DG44 cells differ from the CHO-S^® ^cells with increases in: ALG2, ALG3, ALG9 and ALG12 (mannosyltransferases), ALG8 and ALG10 (glucosyltransferases), ALG14 (acetylglucosaminyltransferase), and B4GALT5 (galactosyltransferase). These increases in gene expression in DG44 cells seem to coincide with the higher galactosylation profiles observed in the glycan analysis.

## Conclusions

Differences in growth, titer and glycoform distribution were observed between CHO-S^® ^and CHO DG44 cells. DG44 cells had higher expression of glycosylation transferase genes compared to CHO-S^® ^cells. Components A and B had synergistic effects on terminal galactosylation (Figure 1), showed no changes in gene expression and could be acting as cofactors/substrates with glycosylation enzymes.

